# Sex-Specific Impact of 17β-Estradiol and Testosterone Levels on Inflammation and Injury in Acute Myocardial Infarction—Preliminary Results

**DOI:** 10.3390/biomedicines13061466

**Published:** 2025-06-13

**Authors:** Niya E. Semerdzhieva, Adelina D. Tsakova, Vesela V. Lozanova

**Affiliations:** 1 Clinic of Internal Medicine, University Emergency Medicine Hospital ‘Pirogov’, 1606 Sofia, Bulgaria; 2 Department of Clinical Laboratory, Medical University, 1431 Sofia, Bulgaria; 3 Clinical Laboratory, University Hospital ‘Sofiamed’, 1797 Sofia, Bulgaria; adelina_d@abv.bg; 4 Deparment of Chemistry and Biochemistry, Medical University, 1431 Sofia, Bulgaria; vlozanova@medfac.mu-sofia.bg

**Keywords:** 17β-estradiol, total testosterone, oxidized low-density lipoproteins, C-reactive protein, acute myocardial infarction, ventricular tachycardia

## Abstract

**Background:** Estrogens play a protective role during the early stages of life. However, endogenous 17β-estradiol (E2) can accelerate atherosclerosis progression. Aim: The purpose of this study was to test for the significance of the sex-specific associations of gonadal hormones with the extent of the inflammatory response, myocardial damage, and ventricular arrhythmia risk in acute myocardial infarction (MI). **Materials and Methods:** Study design: single-center cohort study. Blood samples for the assessment of sex steroids (E2, total testosterone [T]), oxidized low-density lipoproteins, high-sensitivity C-reactive protein (CRP), white blood cell (WBC) counts, and cardiac enzymes were collected 48 h after the onset of symptoms (and within 6 h after PCI) from 111 patients (37% women) with acute MI. Coronary disease severity, left ventricular systolic function (LV), and indices of ventricular repolarization were assessed using coronary angiography, echocardiography, and a conventional electrocardiogram, respectively. **Results:** In men with acute MI, peak cardiac enzyme levels were predicted by post-percutaneous coronary intervention (PCI) E2 plasma levels, peak WBC count, and peak CRP plasma levels. T levels and the E2/T ratio were associated with post-PCI CRP in these men. For women, peak WBC count was a marker of highest testosterone, and only WBC count was a significant indicator of myocardial injury extent. The incidence of acute ventricular tachycardia detected in AMI was significantly associated with left ventricular ejection fraction and with peak WBC count (as a tendency) regardless of sex. A longer duration of cardiac repolarization prior to PCI was predicted by lower ejection fractions in men and by age, CRP, and testosterone levels in female patients. **Conclusions:** During acute MI, elevated endogenous estradiol levels in men and increased leukocytes in women indicate acute myocardial damage. Post-PCI plasma inflammatory markers are sex-specific confounding factors for acute endogenous E2 levels, T levels, and the E2/T ratio. LV systolic function in men and, characteristically, the acute inflammatory response and testosterone levels in women are predictors of longer ventricular repolarization and arrhythmia risk.

## 1. Introduction

Low plasma concentrations of endogenous 17β-estradiol in early life correlate with an atherogenic lipid profile and endothelial dysfunction in men [1,2], as well as with an increased risk of cardiovascular disease and, in particular, acute myocardial infarction (AMI) in women [3,4]. Furthermore, diseases characterized by hyperandrogenemia in women (e.g., polycystic ovary syndrome) and hypogonadism (low testosterone) in young men are predisposing factors for premature and extensive coronary artery disease [5,6].

In contrast, in cohorts with established cardiovascular disease, high serum estradiol levels in both men and women [7,8] and low serum testosterone in men predict the severity of coronary atherosclerosis [7,9]. The ratio of endogenous estradiol to testosterone (E2/T)—used as a surrogate marker of aromatase activity—indicates an adverse prognosis in older women with known cardiovascular disease [10]. An allele variant of the enzyme aromatase converts testosterone to estradiol in various tissues and is a risk indicator for mortality in men with acute coronary syndrome [11]. Aromatase activity varies throughout life, especially during acute illnesses (e.g., acute myocardial infarction), leading to increased endogenous estradiol concentrations [10,12,13,14]. The clinical implications of these fluctuations in endogenous sex hormone concentrations and aromatase activity in acute coronary disease remain uncertain.

Animal research indicates that bilateral ovariectomy increases ischemia–reperfusion (MI/R) injury of the myocardium. Estrogen treatment can mitigate this by inhibiting endoplasmic reticulum stress, reducing cardiomyocyte apoptosis, and resulting in smaller infarcts [15,16]. However, animal models do not adequately represent the extent of atherosclerosis in older humans, limiting their relevance. Studies involving human participants suggest that men with low serum testosterone levels undergoing primary percutaneous coronary intervention (PCI) for MI with ST elevation experience inferior myocardial reperfusion and myocardial systolic function [17]. Conversely, elevated endogenous estradiol levels independently correlate with the risk of no-reflow in postmenopausal women [18].

The regional inhomogeneities in ventricular refractoriness have been demonstrated to increase during myocardial ischemia and to decrease with the reversal of the ischemic state following successful angioplasty [19]. Higher values of dispersion in ventricular repolarization are related to an increased incidence of ventricular arrhythmias and nonfatal myocardial infarction [19]. In general, the sex-related differences in repolarization duration and dispersion can be explained by the cellular effects of sex steroids on action potential duration and autonomic control of the heart rate [20,21].

Our hypothesis is that the change in endogenous sex hormone concentrations and aromatase activity in acute MI is associated with myocardial damage, the inflammatory response to injury in the myocardium, and ventricular repolarization.

### Purpose

The purpose of this study was to test for the significance of the sex-specific associations of gonadal hormones with the extent of the inflammatory response, myocardial damage, and ventricular arrhythmia risk in acute myocardial infarction (MI).

## 2. Materials and Methods

This single-center cohort study included 111 patients (37% women) diagnosed with AMI and admitted to the Clinic of Cardiology, University Hospital “Alexandrovska,” Sofia, between July 2011 and December 2013. Blood samples were drawn 48 h after symptom onset to measure the levels of sex steroids (total 17β-estradiol [E2], total testosterone [T], dehydroepiandrosterone sulfate), oxidized low-density lipoproteins (oxLDL), high-sensitivity C-reactive protein (hsCRP), white blood cell (WBC) counts, and cardiac enzymes (creatine kinase [CK], Muscle–Brain fraction of CK [CPK-MB], and high-sensitivity troponin T [hsTnT]). To measure coronary disease severity, we calculated the SYNTAX score for each patient with angiographically defined coronary atherosclerosis. The levels of E2 and T, as well as those of cardiac enzymes and inflammatory markers (CRP and WBC), were measured within six hours of PCI. In the group without catheter revascularization, hormones, enzymes, and inflammatory markers were measured within 48 h of symptom onset. If re-evaluated, the highest values were used for analysis. All the patients underwent standard echocardiography. Echocardiographic studies were performed in accordance with the recommendations of the American Society of Echocardiography and the European Association of Cardiovascular Imaging using the Sonos 5500 (Hewlett-Packard, Palo Alto, CA, USA) and Aloka ProSound 10 (Hitachi Aloka Medical, Hitachinaka, Japan) ultrasound systems. LV ejection fraction (EF) was measured using Simpson’s method.

We compared the E2 and T of the patients with AMI with the E2 and T of 15 men and women with stable coronary disease in order to test the hypothesis that endogenous E2 and T plasma concentrations change in the acute phase of myocardial infarction in parallel with the levels of inflammatory markers. Eighteen men and women of similar age as the studied cohort with coronary disease excluded via coronary angiography served as the control group.

Patients diagnosed with secondary hypogonadism or diseases of the adrenal and pituitary glands were excluded. Other exclusion criteria included acute infectious disease, chronic inflammatory disease, known or suspected neoplastic processes, surgical procedures, and trauma within two weeks before hospital admission. The participants had not used hormone or immunoreactive therapies six months before or during this study.

We adhered to the Declaration of Helsinki and received approval from the ethics committee of the Medical University of Sofia. All the participants provided written informed consent. This study was retrospectively registered in the UK’s Clinical Study Registry (ISRCTN) with study registration number ISRCTN62480360.

After a 12 h fast, venous blood samples were collected into EDTA sample tubes, centrifuged at 12,000 rpm for 20 min, and stored at −20 °C until analysis. The hsCRP concentrations were determined using a latex-enhanced immunoturbidimetric assay (Roche Diagnostics GmbH, Manheim, Germany) on the COBAS INTEGRA 700 analyzer. We assessed the levels of steroid hormones and hsTNT using an electrochemiluminescent immunoassay with Roche Diagnostics reagents on the Elecsys 2010 analyzer. These methods have been detailed elsewhere [22,23]. Plasma levels of oxLDL were quantified using the OxiSelect Human Oxidized LDL immunosorbent assay (ELISA; MDA-LDL) kit (Cell Biolabs, San Diego, CA, USA) and a sandwich ELISA [24].

### 2.1. ECG Analysis

Standard 12-lead ECGs were recorded at a paper speed of 25 mm/s and a gain of 10 mm/mV. The QT interval was measured using a standardized technique and corrected with Bazett’s formula. Corrected QT dispersion was calculated as the difference between the maximum and minimum corrected QT intervals [19] assessed using surface body electrocardiography (ECG).

### 2.2. Statistical Analysis

We checked variable distributions using the Kolmogorov–Smirnov and Shapiro–Wilk tests. We explored the associations between variables using both parametric (independent-samples *t*-test) and non-parametric (χ^2^ test, Fisher’s exact tests, and Mann–Whitney U test) methods, further validated by Cox proportional regression and univariate and multivariate analyses. The multivariable model which explored the predictors of the highest cardiac enzyme levels included estradiol, testosterone, the estradiol-to-testosterone ratio, white blood cell count, C-reactive protein, the extent and severity of coronary atherosclerotic plaques measured using the Syntax score, and age. The multivariable model testing the significance of the predictors of the highest levels of hormones and E2/T included white blood cell count, C-reactive protein, the extent and severity of coronary atherosclerotic plaques measured using the Syntax score, left ventricular (LV) ejection fraction (EF) as a measure of LV systolic function, and the age. The analyses were conducted using IBM SPSS Statistics for Windows, Version 19.0. (IBM Corp: Armonk, NY, USA). A two-tailed *p*-value less than 0.05 was deemed significant.

## 3. Results

**Figure 1:**
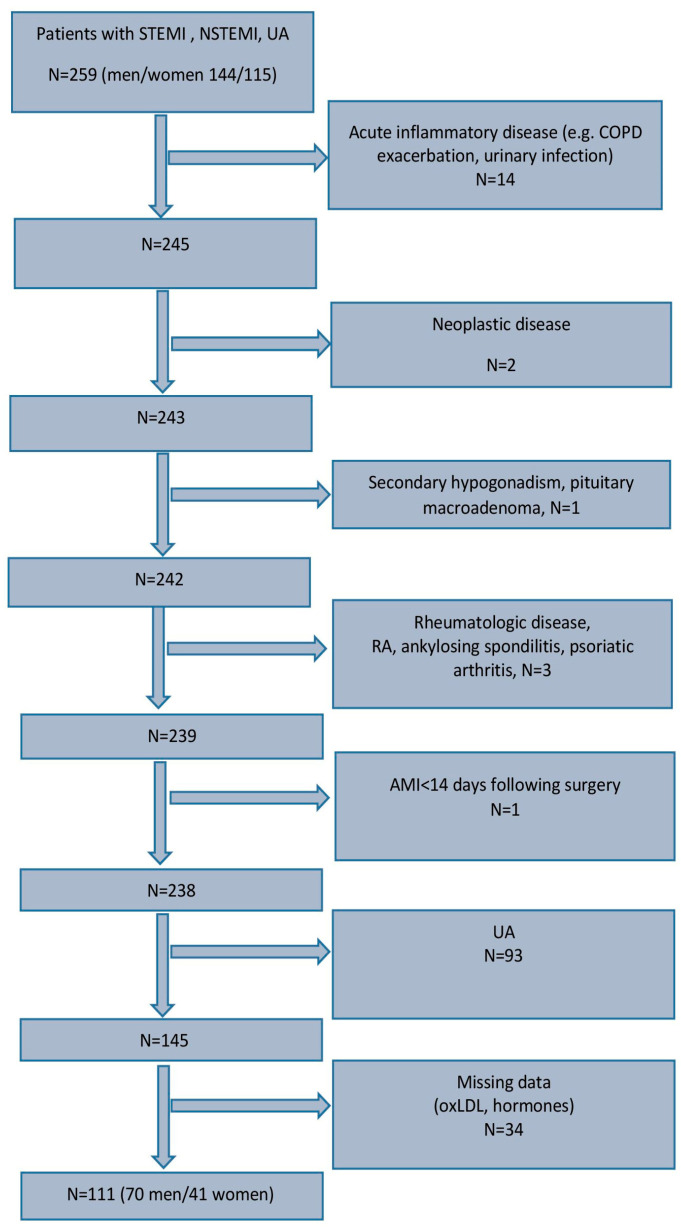
Patient group; inclusion and exclusion criteria. Abbreviations: STEMI, ST elevation myocardial infarction; NSTEMI, myocardial infarction without ST elevation; UA, unstable angina; COPD, chronic obstructive pulmonary disease; RA, rheumatoid arthritis; AMI, acute myocardial infarction; oxLDL, oxidized low-density lipoprotein.

A flowchart showing the steps in the enrollment of the patients studied is presented in Figure 1.

Female patients were, on average, older than male patients (Table 1) and had lower C-reactive protein (CRP) and cardiac enzyme levels. During the acute phase of MI, E2 levels were higher in male patients, while the E2-to-T ratio (E2/T) was lower in female patients (Table 1). The groups did not differ substantially according to MI localization or atherosclerotic involvement of coronary arteries. Women were leaner than men but of similar BMI (Table 1).

A total of 86% of the patients underwent PCI. Among the remaining 14%, some were diagnosed with non-obstructive coronary disease and were suitable for optimal conservative treatment, and the rest of these patients were diagnosed with three- and multi-vessel disease. The latter group was transferred for aorto-coronary by-pass surgery.

Plasma concentrations of E2 and T were higher in the patients with AMI compared to those with stable coronary disease, except there were no differences in E2 between the men with AMI and men with stable CAD (Table 2). E2 levels were higher and T was lower in male patients with AMI compared to the men in the control group (Table 2). Vice versa associations in E2 and T levels in the women with AMI versus female controls were found (Table 2).

**Figure 2:**
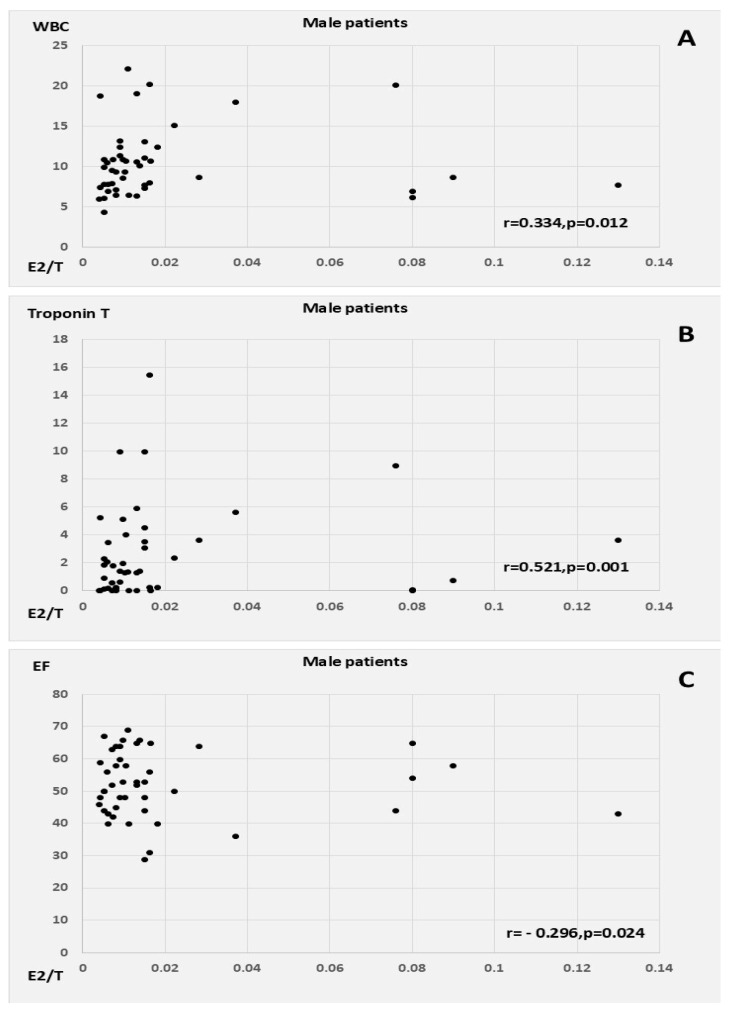
Correlation of E2/T with (**A**) WBC count, (**B**) troponin, and (**C**) ejection fraction in male patients. Abbreviations: WBC, white blood cell; E2/T, ratio of plasma 17β-estradiol to total testosterone concentrations; EF, ejection fraction.

**Figure 3:**
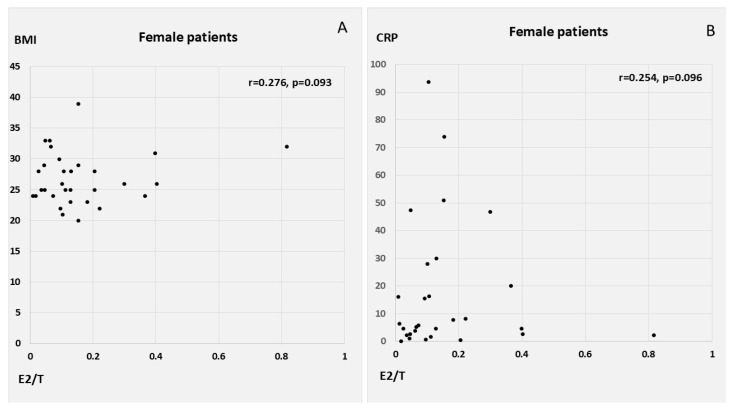
Correlation of E2/T with with heart rate, (**A**) BMI and (**B**) CRP in female patients. Abbreviations: CRP, C-reactive protein; BMI, body mass index; E2/T, ratio of plasma 17β-estradiol to total testosterone concentrations.

E2 correlated positively with CRP, WBC count, Syntax score, and QTc in the male patients with AMI, as well as with T, oxLDL, and MI size (peak cardiac enzymes) in the female AMI patients. E2 had a similar inverse correlation with LVEF in both men and women, in addition to showing a weak tendency for inverse association with age in women. T was associated with WBC count, myocardial injury, and lower EF in the female patients. E2/T was related to peak WBC count, peak troponin levels, and low EF among the male patients with AMI; conversely, in the female patients, E2/T was related to higher heart rates at admission and to a tendency towards higher BMI and CRP (Table 3, Figure 2 and Figure 3).

**Figure 4:**
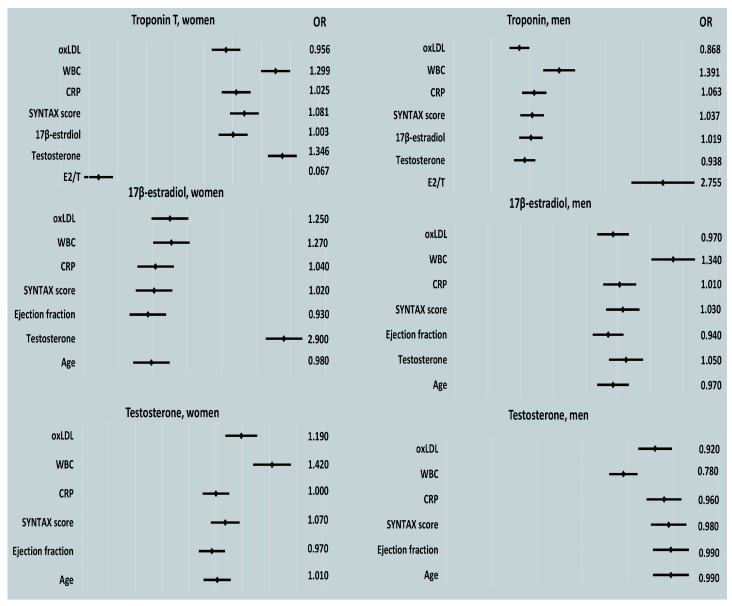
Indicators of troponin T and gonadal steroid hormones in male and female patients assessed using univariable regression analysis. Abbreviations: oxLDL, oxidized low-density lipoprotein; WBC, white blood cell; CRP, C-reactive protein; EF, ejection fraction; T, total testosterone; E2/T, ratio of plasma 17β-estradiol to total testosterone concentrations.

In the male patients, post-PCI E2, CRP, and WBC counts were predictors of peak cardiac enzyme levels, as determined by a univariable regression analysis. For the female patients, peak WBC count was a significant indicator of myocardial injury (Table 4, Table 5 and Table 6 and Figure 4).

For the male patients, high plasma concentrations of E2 and CRP were significant markers for an increase in Troponin T (TnT) levels. Peak WBC count was notably associated with the highest activities of both CK-MB and hs TnT in a multiple regression model (Table 7).

An analysis of the correlations between gonadal steroid hormones and the E2/T ratio during the acute phase of MI revealed that increases in E2 were predicted by increases in CRP in both men and women. The lowest EFs tended to indicate women with the highest E2 levels in the acute phase of MI (Table 8, Figure 4).

In the multivariable model, neither EF nor the maximal elevation of CRP independently predicted peak levels of E2 in the female patients with AMI (EF 0.960, OR 0.890–1.050, *p* = 0.379; CRP 1.040, OR 0.990–1.090, *p* = 0.159).

CRP level and WBC count were tendency markers of a decrease in the plasma levels of total T in the male patients, while WBC count was inversely associated with T in the women with AMI (Table 9). Among the male patients with AMI, peak CRP count showed a trend toward marking the lowest T levels in multivariable analysis (CRP 0.940, OR 0.880–1.000, *p* = 0.053; WBC 1.210, OR 0.700–2.080, *p* = 0.501).

Additionally, a trend suggested that high CRP levels were associated with a higher E2/T ratio (Table 10).

E2 and T were not significantly related to extremely short or long repolarization periods after PCI in either men or women early in the course of MI in this analysis of data. The highest versus the lowest duration of cardiac repolarization at admission showed a relationship with the severity of coronary atherosclerosis, the extent of myocardial injury, and myocardial infarction size (reflected by the levels of cardiac enzymes), as well as with LV systolic function in the subset of male patients with myocardial infarction (Table 11).

Lower ejection fractions of the left ventricle were independently predictive of prolonged repolarization, specifically for the male patients (EF: OR 0.810, 95% CI 0.650–1.010, *p*-0.0059; Syntax score: OR 1.030, *p =* 0.737; CK: OR 1.000, *p =* 680; CK-MB: OR 1.030, *p =* 0.243; troponin T OR 1.000, *p =* 0.998).

We found a significant, non-sex-specific association between the incidence of ventricular tachycardia (VT, 6.8%, n = 7) and left ventricular ejection fraction (43.6% ±12% vs. 53.2% ± 10.7%, *p =* 0.018), as well as a tendency with peak WBC count levels (9.7 × 10^9^/L ± 3.7 × 10^9^/L, *p =* 0.088), during the first week of AMI. Gonadal steroids were not associated with the incidence of VT in an analysis not adjusted for biological sex (E2: 125.9 ± 72.9 pmol/L vs. 131 ± 98.6 pmol/L, *p =* 0.886; T: 9.1 ± 5.6 nmol/L vs. 8.7 ± 7.7 nmol/L, *p =* 0.879; E2/T 0.49 ± 1.32 vs. 0.12 ± 0.3, *p =* 0.975).

## 4. Discussion

We found that during the acute phase of MI, the highest levels of endogenous estradiol and CRP were significantly related to the severity of cardiomyocyte necrosis (as evidenced by peak plasma TnT levels) in the male patients. Conversely, peak WBC count emerged as a sex-specific marker for acute myocardial damage in the women. Interestingly, peak plasma T in the women correlated with the rise in WBC count, while in the men with AMI, T was inversely related to the increase in CRP levels. Furthermore, a positive association was observed between CRP and the E2/T ratio in the male patients.

### 4.1. Sex-Specific Relationship of oxLDL and CRP with E2 and E2/T

According to basic research reports, oxLDL influences estradiol and testosterone production [25,26]. OxLDL, or oxidized low-density lipoprotein, refers to LDL particles that have been modified through oxidation. Oxidized LDL levels can increase after percutaneous coronary intervention, particularly in the acute phase following an MI. The degree of myocardial ischemia acts as a severe cellular stressor, resulting in oxidative stress with increased free radical generation and oxLDL formation. This increase is often transient and may be related to the mechanical disruption of plaque and the release of oxidized lipids during the procedure. However, no significant association of plasma oxLDL levels with inflammatory markers and E2 could be detected in our study, either due to the rapid immune clearance of oxLDL [27], due to the blood samples being obtained too early, or because of a complex relationship which remained statistically undetectable in our small study group.

Many observational studies highlight the significance of the association between gonadal steroid hormones and markers of ongoing low-grade vascular inflammation in atherosclerosis in male patients with chronic coronary disease [7]. Under nonacute conditions, excess adipose infiltration with macrophages leads to increased aromatase expression and elevated endogenous estradiol levels [28]. Obesity might induce the production of chemokine ligand 2 (an MCP-1 receptor) in adipose tissue, thereby inducing inflammation and local aromatase, and subsequently amplifying estrogen levels [29]. A relationship between certain cytokines (IL-6 and TNF-α) and E2 has been documented in male patients with metabolic syndrome [30]. Notably, pro-inflammatory cytokines, such as tumor necrosis factor-alpha (TNF-α), induce the expression of the human aromatase gene in mammary adipose tissue [31].

Recent observational studies show that the effect of exogenous estrogen (hormone replacement therapy) is a predisposing factor for adverse cardiovascular events in women who have had menopausal status for more than ten years [32]. Experimental studies indicate that when atherosclerotic lesions are present, the altered expression of estrogen receptors on the vascular wall further exacerbates arterial wall damage [32,33]. In a low-grade inflammation state, such as established atherosclerosis, E2 can destabilize atherosclerotic plaques by inducing molecules like MCP-1 and matrix metalloproteinases (specifically MMP-2 and MMP-9) in endothelial cells [32]. E2 induces different effects regarding atheromatous plaque instability through different ERs. The overexpression of ERα may result in E2-induced plaque instability by increasing MCP-1 protein expression and MMP-2 activity [32]. In the absence of an inflammatory state, E2, at low concentrations, exerts atheroprotective effects, mainly by decreasing MMP-2 activity and increasing LOX expression via ERβ [32].

During acute illness, a surge in aromatase in response to an elevated production of pro-inflammatory cytokines has been described [34]. A critical illness produces endocrine responses, such as hypogonadotropic hypogonadism in male patients, associated with low luteinizing hormone, LH [35]. Also, an increase in adipose aromatase content, decreased serum T levels, and a rise in estrone levels, with a trend toward a rise in E2, has been observed in both men and women [36]. Aromatase expression in adipose tissue is primarily under the control of its promoter, which is regulated by cytokines and the stress-induced glucocorticoid cortisol [36].

Importantly, several human studies have reported fluctuations in E2 and testosterone levels during acute coronary syndrome [12,13]. Persistently elevated E2 and declining testosterone have been associated with changes in hemostatic factors (notably, increased plasminogen activator inhibitor-1, PAI-1) in men with AMI [12]. Elevated cardiac PAI-1 post-myocardial infarction may contribute to tissue remodeling and augmented cardiac fibrosis [37], creating a prothrombotic profile in male AMI patients and in situ microthromboses.

### 4.2. Relationship of WBC Count with TnT in Female Patients

For the male AMI patients, the peak systemic inflammatory mediators predicted plasma 17β-estradiol levels and E2 correlated with infarct size, while testosterone levels did not emerge as a significant marker of myocardial injury. In contrast, WBC count was associated with T levels and myocardial injury severity specifically in the group of female patients.

According to observational data, in middle-aged women, a higher WBC count indicates an underlying inflammatory state associated with obesity, hyperandrogenemia, and polycystic ovary syndrome (PCOS) [38]. Moreover, testosterone has been directly linked to increased platelet thromboxane A receptor density and maximal platelet aggregation [39]. In particular conditions in which tissue factor biological action is crucial, such as acute myocardial infarction, the neutrophil count increases substantially [40]. Tissue factor is expressed by endothelial cells, platelets, and neutrophils [40]. Inflammatory molecules such as P-selectin and TNF-α share the ability to induce tissue factor synthesis on the neutrophil surface [40]. A testosterone-rich environment may intensify platelet–leukocyte interactions, potentially leading to increased fibrin deposition and extensive coronary thrombus formation, which could be a potential source of distal embolization. Testosterone’s associations with thrombin generation parameters (like fibrinogen, factor VIIc, PAI-1) have been documented in young women with PCOS [41] and middle-aged women [42].

### 4.3. Lack of Overt Relationship of E2 with Myocardial Injury in Women with AMI

Interestingly, only E2 emerged as a predictor of myocardial injury in our study, and specifically in the men. In contrast, for the female AMI patients, troponin levels were associated with higher WBC counts. Notably, earlier population-based studies have proved that the plasma concentration of estradiol and its production rate were significantly higher in healthy men than in age-matched postmenopausal women without cardiovascular disease [43]. The levels of E2 and T measured in our study coincided in time with peak plasma levels of inflammatory and myocardial injury markers (measured within six hours of PCI). Also, E2 and E2/T were significantly associated with the inflammatory response in the acute phase of MI and, in the case of E2, also with myocardial damage, suggesting a potential significance of E2 as a male-specific marker of myocardial injury in small AMI cohorts.

### 4.4. Lack of Significant Association of E2/T and E2 with Body Mass Index

Most E2 production in men arises from the extragonadal aromatization of adrenal androgens and testosterone in muscle and adipose tissues. The rate of testosterone conversion to estradiol is lower than that of androstenedione aromatization to estrone [10,44]. However, the estradiol/testosterone ratio is accepted as a measure of the aromatization of T into E2 in large studies of male patients [45]. The aromatization of C19 steroids in peripheral tissues (adipose and skin fibroblasts) by enzyme aromatase is the primary mechanism for estrogen formation in postmenopausal women [10]. The androstenedione-to-estrone ratio is used as a marker of aromatase enzyme activity in postmenopausal women [10]. Nevertheless, the median value of estrone-to-androstenedione concentrations correlated significantly with the estradiol/testosterone ratio in postmenopausal women [10].

Our analysis revealed that body mass index (BMI) did not significantly correlate with the levels of gonadal steroid hormones, E2/T, or inflammatory markers. Other studies have emphasized a stronger relationship between abdominal adipose tissue thickness (rather than BMI) and the levels of inflammatory mediators and sex hormones [46]. Inflammatory pathways are activated soon after high-fat diet consumption, leading to inflammatory macrophage accumulation in white adipose tissue even before overt obesity manifests [47]. The association of sex hormones and inflammatory markers with BMI as a measure of overall adiposity is obviously far too weak compared to the correlation of abdominal adipose tissue thickness with inflammatory mediators and sex hormones.

### 4.5. Association of QTc with EF in Male Patients and of QTc with CRP and Testosterone in Female Patients

The incidence of ventricular tachycardia detected until the end of the first week of AMI was non-sex-specifically associated with left ventricular ejection fraction and, as a tendency, with peak WBC count. In the male patients, the longer duration of cardiac repolarization (QTc) prior to PCI was related to the severity of coronary disease, infarction size (peak cardiac enzymes), and EFs. Specifically in the male patients, the reduction in LV systolic function showed a significant independent relationship with longer QTc in the multivariable model. In the subset of female patients with AMI, QTc showed a trend for association with younger age, lower CRP, and higher total testosterone levels but was not independently predicted by any of these variables. These results showed that regarding the women with acute MI, factors, such as higher endogenous plasma T levels, might be implicated in the predisposition to ventricular arrhythmia via mechanisms not directly involving acute myocardial injury.

Among the possible mechanisms involved in the deleterious actions of supraphysiological doses of testosterone in female individuals are unfavorable changes in the lipid profile, procoagulatory effects, activation of the sympathetic nervous system and renin–angiotensin–aldosterone system, and insulin resistance. These indirect actions of testosterone can affect the vasculature. Among its direct effects, the stimulation of pro-inflammatory enzymes in the vasculature [e.g., thromboxane synthase, cyclooxygenase-1 (COX-1), and COX-2] and reactive oxygen species generation in vascular smooth muscle cells may decrease nitric oxide (NO) bioavailability and lead to increased vasoconstriction, increased blood pressure, and renal dysfunction [48].

In women, low testosterone levels may be linked to an increased risk of ventricular arrhythmias, including sudden cardiac death by prolonging ventricular repolarization [48]. However, oxidative stress can also elevate the risk of arrhythmias in both sexes. Testosterone upregulates the expression of NADP oxidase (NOX4) and increases oxidative stress [48]. Increased levels of mitochondrial NOX4 during aging lead to a higher inducibility of self-limiting ventricular arrhythmias [49]. Transgenic mice with mitochondria-targeted Nox4 overexpression showed a significantly higher incidence of pacing-induced VT, associated with shorter action potential duration due to increased transient outward potassium currents. Fractional sarcoplasmic reticulum Ca^2+^ release and Ca^2+^ leak remained intact despite these changes [49].

### 4.6. Significance and Limitations

The results of this study contribute to scientific theory beyond the pathophysiology of coronary disease. Incomplete gonadal hormone suppression is involved in resistance to adjuvant therapy in different hormone-sensitive neoplasms (breast cancer and prostate cancer). The knowledge that variations in endogenous 17β-estradiol, testosterone, and E2/T associated with systemic increases in inflammatory markers can occur in pathologic settings (e.g., acute myocardial infarction) is important for discerning resistant malignancies and for developing optimal protocols for therapy, including adjuvant antitumor therapy.

This study has some limitations. First, the hormone levels were measured once. The changes in hormone levels after the infarction are likely to better characterize the observed relationships of inflammatory markers with E2 and E2/T. Second, estrone levels are higher than estradiol in postmenopausal women, and the ratio of androstenedione to estrone (not the E2/T ratio) directly characterizes aromatase activity. Third, we did not have any measurement of fat mass except BMI. The thickness of abdominal adipose tissue measured by ultrasound indicates the effects of obesity with higher accuracy. Finally, in up to 14% of the patients, PCI was not performed, mainly because of non-obstructive coronary disease. In these patients, the peak levels of cardiac enzymes, and thus the size of the infarction, may not have been precisely evaluated due to the blood samples being obtained too early.

## 5. Conclusions

During acute MI, elevated endogenous estradiol levels in men and increased leukocytes in women indicate acute myocardial damage. Post-PCI plasma inflammatory markers are sex-specific confounding factors for acute endogenous E2 levels, T levels, and the E2/T ratio. LV systolic function in men and the acute inflammatory response and testosterone levels characteristically for women are predictors of longer ventricular repolarization and arrhythmia risk.

## Figures and Tables

**Table 1:** Patient characteristics compared by sex.

Variables	Men (n = 70)	Women (n = 41)	*p*-Value
Age, years	62.8 ± 12.7	70.7 ± 10	0.001
Hypertension, n %	66 (94)	40 (97)	NS
Dyslipidemia, n %	58 (83)	38 (93)	NS
Diabetes mellitus, n %	24 (34)	18 (43)	0.320
oxLDL, mg/mL	10.4 ± 7.3	8.1 ± 4.9	0.099
CRP, mg/L	27.7 ± 52	16.9 ± 21.7	0.032
WBC, ×10^9^ L	10.3 ± 3.8	9.8 ± 3.3	0.662
STEMI, n %	38 (34)	19 (17)	1.000
NSTEMI, n %	31 (28)	22 (19)	
Anterior MI, n %	28 (25)	22 (19)	0.527
Inferior MI, n %	22 (19)	12 (12)	
Lateral MI, n %	12 (11)	4 (4)	
Non-obstructive CAD, n %	2 (2)	3 (2)	0.519
1-vessel CAD, n %	15 (13)	12 (11)	
2-vessel CAD, n %	28 (26)	13 (12)	
3-Vessel CAD, n %	23 (21)	14 (10)	
Syntax score	14.1 ± 10.6	11.2 ± 9.2	0.027
EF, %	53.6 ± 10	52.4 ± 12.9	0.606
BMI, kg/m^2^	28.4 ± 4.3	27.4 ± 5.3	0.330
Weight, kg	85.9 ± 13.6	67.6 ± 10	<0.0001
CK, U/L	1178.9 ± 1486.4	474 ± 722.4	0.001
CK-MB, U/L	116.1 ± 146	63.6 ± 82.1	0.057
hsTnT, ng/mL	2.5 ± 3.4	1.4 ± 2.5	0.036
E2, pmol/L	155.8 ± 69.6	108.7 ± 126.9	<0.0001
T, nmol/L	13.4 ± 5.2	1.7 ± 3	<0.0001
E2/T	0.02 ± 0.03	0.23 ± 0.51	<0.0001
β-blocker, n %	19 (27)	18 (36)	0.571
ACE-I/ARB, n %	18 (41)	23 (59)	0.197
Statin, n %	1 (2)	2 (5)	NS
Antiplatelet, n %	22 (32)	13 (32)	1.000

Abbreviations: oxLDL, oxidized low-density lipoprotein; CRP, C-reactive protein; WBC, white blood cell; EF, ejection fraction; BMI, body mass index; CK, creatine kinase; CK-MB, MB fraction of CK; hsTnT, high-sensitivity troponin T; E2, endogenous 17β-estradiol; T, total testosterone; E2/T, ratio of plasma 17β-estradiol to total testosterone concentrations.

**Table 2:** Age, E2, and T in patients with AMI, patients with stable CAD, and controls.

Variables	Men		*p*-Value	Women		*p*-Value
	**AMI**	**Stable CAD,** **n = 15**		**AMI**	**Stable CAD,** **n = 15**	
Age	62.8 ± 12.7	61.8 ± 14	0.413	70.7 ± 10	74.6 ± 6.4	0.106
E2	155.8 ± 69.6	115.4 ± 33.4	0.007	108.7 ± 126.9	66.3 ± 29.6	0.067
T	15.7 ± 14.7	14.1 ± 8.4	0.875	1.7 ± 3.0	0.8 ± 0.4	0.088
	**AMI**	**Control,** **n = 18**		**AMI**	**Control,** **n = 18**	
Age	62.8 ± 12.7	59.1 ± 13.6	0.179	70.7 ± 10	57.4 ± 15.2	0.050
E2	155.8 ± 69.6	139.2 ± 115.9	0.0001	108.7 ± 126.9	223.7 ± 255.4	<0.0001
T	13.4 ± 5.2	19 ± 9.6	<0.0001	1.7 ± 3	0.6 ± 0.3	<0.0001

Abbreviations: AMI, acute myocardial infarction; CAD, coronary disease; E2, endogenous 17β-estradiol; T, total testosterone.

**Table 3:** E2, T, and E2/T and patient characteristics–correlation analysis.

Patients	Male Patients						Female Patients					
Variables	E2		T		E2/T		E2		T		E2/T	
	r	*p*	r	*p*	r	*p*	r	*p*	r	*p*	r	*p*
T	0.100	0.457					0.345	0.022 *				
BMI	0.087	0.524	0.157	0.248	−0.207	0.123	−0.174	0.297	−0.187	0.262	0.276	0.093 *
Age	−0.043	0.749	−0.103	0.443	0.156	0.242	−0.259	0.094 *	−0.077	0.625	0.096	0.542
oxLDL	−0.010	0.947	−0.093	0.544	0.040	0.793	0.444	0.004 *	0.063	0.697	0.130	0.424
CRP	0.398	0.002 *	−0.340	0.009 *	0.021	0.874	0.488	0.001 *	0.139	0.370	0.254	0.096 *
WBC	0.351	0.008 *	−0.253	0.060 *	0.334	0.012 *	0.299	0.051 *	0.459	0.002 *	−0.195	0.210
Syntax score	0.303	0.021 *	−0.139	0.299	0.156	0.243	0.238	0.125	0.050	0.750	−0.109	0.485
EF	−0.237	0.072 *	0.003	0.983	−0.296	0.024 *	−0.434	0.003 *	−0.439	0.003 *	−0.009	0.951
CK	0.182	0.171	0.018	0.898	0.127	0.335	0.226	0.146	0.776	0.001 *	−0.158	0.307
CK-MB	0.198	0.136	−0.001	0.994	0.107	0.425	0.334	0.027 *	0.612	0.001 *	−0.171	0.267
hsTnT	0.333	0.111	−0.158	0.238	0.521	0.001 *	0.036	0.059 *	0.320	0.029 *	−0.137	0.376
Heart rate	−0.018	0.910	−0.314	0.038 *	−0.067	0.666	0.269	0.112	−0.045	0.793	0.457	0.005 *
QTc	0.337	0.020 *	−0.038	0.879	−0.013	0.932	−0.045	0.791	0.164	0.331	−0.178	0.292

Abbreviations: E2, endogenous 17β-estradiol; T, total testosterone; E2/T, estradiol-to-testosterone ratio; BMI, body mass index; oxLDL, oxidized low-density lipoprotein; CRP, C-reactive protein; WBC, white blood cell; EF, ejection fraction; CK, creatine kinase; CK-MB, MB fraction of CK; hsTnT, high-sensitivity troponin T; * denotes significant or as tendency significant associtaions.

**Table 4:** Association of gonadal steroids and inflammatory and oxidative stress markers with peak creatine kinase (CK) activity.

CK	Lowest Tertile	Highest Tertile	*p*-Value	OR	95% CI	*p*-Value
Male patients						
oxLDL	10 ± 8.9	10 ± 7.9	0.317	0.985	0.908–1.070	0.725
WBC	8.4 ± 1.9	11.9 ± 4.4	0.002 *	1.487	1.081–2.045	0.015 *
CRP	11.6 ± 20.5	49.5 ± 56.3	0.005 *	1.040	1.003–1.078	0.033 *
Syntax score	15.9 ± 10.8	17.5 ± 10.2	0.301	1.016	0.958–1.077	0.593
17β-estradiol	133.6 ± 54.4	185.6 ± 92.8	0.015 *	1.011	1.000–1.022	0.047 *
Testosterone	14.5 ± 6.2	13 ± 4.6	0.179	0.947	0.843–1.062	0.352
E2/T	0.016 ± 0.016	0.021 ± 0.017	0.156	6.788	0.154–298.9	0.321
Female patients						
oxLDL	9.6 ± 7.9	10.9 ± 7.3	0.314	1.026	1.026–1.134	0.620
WBC	8.6 ± 2.1	11.2 ± 3.3	0.026 *	1.426	1.017–1.998	0.039 *
CRP	10.9 ± 16.8	22.5 ± 25.8	0.170	1.030	0.985–1.078	0.198
Syntax score	10.7 ± 8.1	13.8 ± 9.9	0.395	1.041	0.951–1.141	0.381
17β-estradiol	105.5 ± 109.1	155.6 ± 178.1	0.377	1.033	0.997–1.009	0.383
Testosterone	0.98 ± 1.57	3.23 ± 4.5	0.050	1.346	0.882–2.054	0.169
E2/T	0.39 ± 0.84	0.18 ± 0.21	0.181	0.446	0.063–3.167	0.419

Abbreviations: oxLDL, oxidized low-density lipoprotein; CRP, C-reactive protein; WBC, white blood cell; CK, creatine kinase; CK-MB, MB fraction of CK; E2, endogenous 17β-estradiol; T, total testosterone; * denotes significant or as tendency significant associtaions.

**Table 5:** Relationship of estradiol (E2), testosterone (T), inflammation, and oxidative stress with post-PCI activity of CK-MB.

CK-MB	Lowest Tertile	Highest Tertile	*p*-Value	OR	95% CI	*p*-Value
Male patients						
oxLDL	10.4 ± 8.3	9.8 ± 7.7	0.794	0.983	0.910–1.074	0.787
WBC	8.2 ± 1.8	12.7 ± 4.7	<0.0001 *	1.709	1.174–2.488	0.005 *
CRP	15.7 ± 27.7	58.4 ± 66.2	0.008 *	1.024	1.002–1.046	0.029 *
Syntax score	15.5 ± 10.2	18.1 ± 9.5	0.370	1.028	0.968–1.092	0.364
17β-estradiol	128 ± 45.9	188.9 ± 90.1	0.003 *	1.018	1.004–1.032	0.013 *
Testosterone	13.8 ± 5.4	12.9 ± 4.5	0.268	0.963	0.856–1.083	0.528
E2/T	0.019 ± 0.021	0.019 ± 0.015	0.470	0.879	0.033–23.517	0.939
Female patients						
oxLDL	8.9 ± 6.6	8.9 ± 3.7	0.993	0.991	0.913–1.076	0.838
WBC	8.3 ± 2.5	11.1 ± 3.3	0.022 *	1.384	1.022–1.875	0.036 *
CRP	11.7 ± 16.3	22.3 ± 25.9	0.195	1.027	0.984–1.072	0.217
Syntax score	13.2 ± 16.3	22.3 ± 25.9	0.195	0.979	0.890–1.076	0.659
17β-estradiol	101.1 ± 106.9	137.9 ± 171.1	0.245	1.002	0.996–1.008	0.489
Testosterone	0.73 ± 0.53	3.1 ± 4.6	0.039	2.115	0.594–7.527	0.248
E2/T	0.36 ± 0.81	0.16 ± 0.20	0.188	0.443	0.057–3.472	0.439

Abbreviations: PCI, percutaneous coronary intervention; oxLDL, oxidized low-density lipoprotein; CRP, C-reactive protein; WBC, white blood cell; CK, creatine kinase; CK-MB, MB fraction of CK; E2, endogenous 17β-estradiol; T, total testosterone. * denotes significant or as tendency significant associtaions.

**Table 6:** Association of hormones and inflammatory and oxidative stress markers with troponin T in acute myocardial infarction (AMI).

Troponin T	Lowest Tertile	Highest Tertile	*p*-Value	OR	95% CI	*p*-Value
Male patients						
oxLDL	12.1 ± 8.8	7.9 ± 3.6	0.630	0.868	0.769–1.625	0.161
WBC	8.6 ± 2.2	12.1 ± 4.4	0.003 *	1.391	1.076–1.768	0.012 *
CRP	11.4 ± 16.6	61.3 ± 63.7	0.001 *	1.063	1.017–1.111	0.006 *
Syntax score	14.6 ± 10.8	17.9 ± 8.9	0.258	1.037	0.974–1.103	0.257
17β-estradiol	123 ± 49.9	195.8 ± 89.7	0.001 *	1.019	1.006–1.033	0.005 *
Testosterone	13.9 ± 5.7	11.8 ± 5.5	0.125	0.938	0.840–1.046	0.250
E2/T	0.018 ± 0.021	0.029 ± 0.035	0.107	2.755	0.178–42.656	0.468
Female patients						
oxLDL	8.4 ± 7.0	8.8 ± 3.7	0.410	0.954	0.879–1.035	0.254
WBC	8.8 ± 2.8	11.8 ± 4.1	0.034 *	1.299	1.022–1.685	0.048 *
CRP	12.1 ± 19	23 ± 25.6	0.198	1.025	0.986–1.066	0.213
Syntax score	8.9 ± 8.6	15 ± 9.5	0.089 *	1.081	0.985–1.186	0.099 *
17β-estradiol	101.6 ± 102.9	153.5 ± 179.2	0.172	1.003	0.997–1.009	0.355
Testosterone	0.98 ± 1.57	3.23 ± 4.5	0.050	1.346	0.882–2.054	0.169
E2/T	0.42 ± 0.81	0.12 ± 0.10	0.178	0.067	0.001–5.665	0.233

Abbreviations: AMI, acute myocardial infarction; oxLDL, oxidized low-density lipoprotein; CRP, C-reactive protein; WBC, white blood cell; E2, endogenous 17β-estradiol; T, total testosterone; * denotes significant or as tendency significant associtaions.

**Table 7:** CRP and E2 as indicators of myocardial injury in male patients with acute myocardial infarction.

Male Patients			
**Troponin T**	**OR**	**95% CI**	***p*** **-Value**
17β-estradiol	1.018	1.001–1.035	0.042 *
CRP	1.048	1.004–1.093	0.031 *
WBC	1.096	0.811–1.480	0.552
**CK-MB**	**OR**	**95% CI**	***p*** **-Value**
WBC	1.503	1.018–2.217	0.040 *
CRP	1.017	0.994–1.040	0.144
17β-estradiol	1.014	0.996–1.032	0.129
**CK**	**OR**	**95% CI**	***p*** **-Value**
WBC	1.301	0.923–1.833	0.133
CRP	1.025	0.992–1.058	0.137
17β-estradiol	1.005	0.992–1.018	0.426

Abbreviations: CRP, C-reactive protein; WBC, white blood cell; CK, creatine kinase; CK-MB, MB fraction of CK; * denotes significant or as tendency significant associtaions.

**Table 8:** Relationship of 17β-estradiol with oxLDL and inflammatory markers.

17β-Estradiol	Lowest Tertile	Highest Tertile	*p*	OR	95% CI	*p*-Value
Male patients						
Age	71.1 ± 14.9	66.1 ± 12.3	0.325	0.970	0.920–1.030	0.313
oxLDL	10 ± 7.4	8.7 ± 5.5	0.625	0.970	0.850–1.100	0.614
WBC	8.5 ± 2.0	11.8 ± 5.1	0.023	1.340	0.980–1.830	0.066 *
CRP	21.9 ± 27.5	45 ± 57.3	0.049	1.010	0.990–1.040	0.209
Syntax score	14.5 ± 10	18 ± 11.4	0.373	1.030	0.960–1.110	0.365
EF	53.5 ± 8.6	48.3 ± 19.6	0.164	0.940	0.860–1.020	0.134
Testosterone	12.6 ± 4.8	13.9 ± 6.4	0.501	1.050	0.920–1.20	0.487
Female patients						
Age	70 ± 7.8	67.8 ± 12.9	0.637	0.980	0.900–1.060	0.618
oxLDL	11 ± 5.8	7.1 ± 3.6	0.089	1.250	0.950–1.640	0.115
WBC	8.6 ± 2.5	10.5 ± 3.3	0.150	1.270	0.920–1.760	0.149
CRP	11 ± 16	51 ± 53.5	0.028	1.040	1.000–1.100	0.067 *
Syntax score	12.2 ± 8.5	14.1 ± 10.5	0.653	1.020	0.930–1.120	0.635
EF	60.3 ± 7.8	47.6 ± 17.8	0.050	0.930	0.860–1.000	0.065 *
Testosterone	0.6 ± 0.5	2.9 ± 3.9	0.065	2.90	0.480–17.620	0.247

Abbreviations: oxLDL, oxidized low-density lipoprotein; CRP, C-reactive protein; WBC, white blood cell; EF, ejection fraction; * denotes significant or as tendency significant associtaions.

**Table 9:** Relationship of total testosterone with oxLDL and inflammatory markers.

Testosterone	Lowest Tertile	Highest Tertile	*p*	OR	95% CI	*p*-Value
Male patients						
Age	62.9 ± 12.1	62.1 ± 11	0.839	0.990	0.980–1.060	0.832
oxLDL	11.2 ± 7.9	8.5 ± 4.2	0.315	0.920	0.770–1.090	0.335
WBC	11.6 ± 4.6	8.7 ± 2.5	0.048 *	0.780	0.590–1.020	0.072 *
CRP	61.2 ± 65.8	10.3 ± 13.3	0.010 *	0.960	0.910–1.000	0.057 *
Syntax score	18.4 ± 9.6	16.3 ± 9.5	0.550	0.980	0.900–1.060	0.539
EF	50.9 ± 10.5	50 ± 8.3	0.804	0.990	0.910–1.070	0.795
Female patients						
Age	69.7 ± 10.8	71.1 ± 12	0.782	1.010	0.940–1.090	0.769
oxLDL	7.5 ± 4.7	9.9 ± 2.9	0.157	1.190	0.930–1.510	0.160
WBC	8.1 ± 2.3	11.4 ± 4.1	0.024 *	1.420	1.010–1.980	0.044 *
CRP	29 ± 37.8	62.7 ± 32.7	0.929	1.000	0.980–1.020	0.925
Syntax score	8.7 ± 8.1	12.8 ± 8.1	0.236	1.070	0.960–1.190	0.227
EF	50.6 ± 10.9	45.7 ± 14.6	0.377	0.970	0.910–1.040	0.360

Abbreviations: oxLDL, oxidized low-density lipoprotein; CRP, C-reactive protein; WBC, white blood cell; EF, ejection fraction; * denotes significant or as tendency significant associtaions.

**Table 10:** Relationship of E2/T with oxLDL and inflammatory markers.

E2/T	Lowest Tertile	Highest Tertile	*p*	OR	95% CI	*p*-Value
Male patients						
Age	69.8 ± 12.7	67.2 ± 11.7	0.522	0.980	0.930–1.040	0.509
oxLDL	8.0 ± 3.2	8.5 ± 4.9	0.699	1.031	0.895–1.202	0.692
WBC	9.7 ± 2.8	10.4 ± 4.3	0.494	1.058	0.902–1.242	0.488
CRP	29.1 ± 46.4	57.2 ± 62	0.062 *	1.010	0.999–1.022	0.079 *
Syntax score	14.3 ± 9.5	17.4 ± 10.6	0.270	1.032	0.976–1.090	0.268
EF	50.3 ± 7.9	49.9 ± 11.5	0.901	1.000	0.930–1.070	0.897
Female patients						
Age	74.8 ± 6.7	70.3 ± 10.9	0.293	0.940	0.840–1.050	0.285
oxLDL	8.6 ± 4.6	8.4 ± 6.7	0.857	1.031	0.884–1.162	0.850
WBC	10.3 ± 3.7	8.4 ± 2.0	0.115	0.781	0.565–1.079	0.133
CRP	11.9 ± 16.5	20.2 ± 24.1	0.302	1.021	0.981–1.063	0.300
Syntax score	11.1 ± 7.1	11.2 ± 10.6	0.980	1.001	0.917–1.093	0.979
EF	55.9 ± 13	52.1 ± 15.3	0.555	0.980	0.920–1.050	0.534

Abbreviations: oxLDL, oxidized low-density lipoprotein; CRP, C-reactive protein; WBC, white blood cell; EF, ejection fraction; * denotes significant or as tendency significant associtaions.

**Table 11:** Relationship of MI size, extent of coronary atherosclerosis, left ventricular systolic function, and hormones with repolarization period (QTc).

QTc	Lowest Tertile	Highest Tertile	*p*	OR	95% CI	*p*-Value
Male patients						
Age	64.8 ± 11.3	63.9 ± 13.9	0.874	1.270	0.800–2.010	0.310
oxLDL	7.8 ± 4.2	11.0 ± 9.0	0.312	1.090	0.920–1.290	0.233
WBC	7.8 ± 1.4	9.8 ± 3.8	0.112	1.360	0.900–2.060	0.142
CRP	14.7 ± 15.3	37.4 ± 63.4	0.240	1.010	0.990–1.040	0.291
Syntax score	11.3 ± 6.1	18.3 ± 12.9	0.099 *	1.090	0.970–1.230	0.099 *
EF	55.8 ± 6.6	48.5 ± 8.3	0.026 *	0.870	0.770–1.000	0.043 *
17β-estradiol	149.6 ± 36	183.7 ± 89.4	0.233	0.920	0.760–1.120	0.419
Testosterone	15.3 ± 5.2	15.5 ± 6.4	0.917	2.750	0.490–15.360	0.248
E2/T	0.02 ± 0.03	0.01 ± 0.01	0.290	0.080	0.001–14.680	0.309
CK	437.8 ± 555.9	1464.3 ± 146.1	0.033 *	1.000	0.970–1.030	0.064 *
CK-MB	49.9 ± 52.1	146.7 ± 144.8	0.040 *	1.010	1.000–1.020	0.077 *
Troponin T	0.82 ± 1.23	2.93 ± 3.1	0.044 *	1.650	0.980–2.770	0.060 *
Female patients						
Age	72 ± 5.9	63.2 ± 10.3	0.045 *	0.910	0.810–1.020	0.118
oxLDL	6.3 ± 3.1	5.4 ± 2.6	0.530	0.890	0.620–1.270	0.510
WBC	8.4 ± 1.9	8.8 ± 2.3	0.679	1.110	0.690–1.800	0.657
CRP	32.7 ± 54.6	5.6 ± 5	0.054 *	0.880	0.740–1.050	0.169
Syntax score	13.3 ± 11.8	7.7 ± 9.6	0.303	1.100	0.950–1.280	0.190
EF	53.4 ± 14.5	54 ± 15.4	0.937	1.000	0.940–1.070	0.932
17β-estradiol	83.8 ± 10.2	80.8 ± 56	0.957	1.000	0.990–1.010	0.932
Testosterone	0.68 ± 0.28	1.33 ± 1.86	0.070 *	11.700	0.600–22.7	0.105
E2/T	0.17 ± 0.17	0.13 ± 0.06	0.502	0.050	0.001–38.9	0.295
CK	405.6 ± 94.3	249.3 ± 281.3	0.460	1.000	1.000–1.000	0.434
CK-MB	61.8 ± 94.3	36.1 ± 37.8	0.434	0.990	0.980–1.010	0.432
Troponin T	1.05 ± 1.52	1.38 ± 3.1	0.768	1.060	0.720–1.560	0.754

Abbreviations: QTc, corrected duration of cardiac repolarization; oxLDL, oxidized low-density lipoprotein; CRP, C-reactive protein; WBC, white blood cell; EF, ejection fraction; E2/T, ratio of endogenous 17β-estradiol to total testosterone; CK, creatine kinase; CK-MB, MB fraction of CK; hsTnT, high-sensitivity troponin T; * denotes significant or as tendency significant associtaions.

## Data Availability

Data is available from the corresponding author; details regarding data supporting the reported results can be sent to editors and reviewers upon request.

## References

[1] Rochira V., Carani C. (2009). Aromatase deficiency in men: A clinical perspective. Nat. Rev. Endocrinol..

[2] Lew R., Komesaroff P., Williams M., Dawood T., Sudhir K. (2003). Endogenous estrogens influence endothelial function in young men. Circ. Res..

[3] Chen I.J., Shoupe D., Karim R., Stanczyk F.Z., Kono N., Sriprasert I., Hodis H.N., Mack W.J. (2023). The association of hysterectomy with or without ovarian conservation with subclinical atherosclerosis progression in healthy postmenopausal women. Menopause.

[4] Lo A.C.Q., Lo C.C.W., Oliver-Williams C. (2023). Cardiovascular disease risk in women with hyperandrogenism, oligomenorrhea/menstrual irregularity or polycystic ovaries (components of polycystic ovary syndrome): A systematic review and meta-analysis. Eur. Heart J. Open..

[5] Birdsall M.A., Farquhar C.M., White H.D. (1997). Association between polycystic ovaries and extent of coronary artery disease in women having cardiac catheterization. Ann. Intern. Med..

[6] Luo Y., Zhou Y., Jiang H., Zhu Q., LV Q., Zhang X., Gu R., Yan B., Wei L., Zhu Y. (2024). Identification of potential diagnostic genes for atherosclerosis in women with polycystic ovary syndrome. Sci. Rep..

[7] Naessen T., Sjogren U., Bergquist J., Larsson M., Lind L., Kushnir M.M. (2010). Endogenous steroids measured by high-specificity liquid chromatography-tandem mass spectrometry and prevalent cardiovascular disease in 70-year-old men and women. J. Clin. Endocrinol. Metab..

[8] Benn M., Voss S.S., Holmegard H.N., Jensen G.B., Tybjærg-Hansen A., Nordesgaard B.G. (2015). Extreme concentrations of endogenous sex hormones, ischemic heart disease, and death in women. Arterioscler. Thromb. Vasc. Biol..

[9] Wranicz J.K., Cygankiewicz I., Kula P., Walczak-Jedrzejowska R., Slowikowska-Hilczer J., Kula K. (2006). Endogenous estradiol and testosterone may predispose toward atherogenic lipid profile, but higher blood level of testosterone is associated with lower number of stenoses in the coronary arteries of men with coronary disease. Int. J. Biomed. Sci..

[10] Laughlin G.A., Ix J.H., Cummins K., Allison M.A.A., Daniels L.B. (2012). Extremes of an aromatase index predict increased 25-year risk of cardiovascular mortality in older women. Clin. Endocrinol..

[11] Wang B., Fu Z., Ma Y., Huang D., Liu F., Dong C., Wang T., Meng Y. (2016). Identification of a CYP19 gene single-nucleotide polymorphism associated with a reduced risk of coronary heart disease. Genet. Test. Mol. Biomarkers..

[12] Pugh P.J., Channer K.S., Parry H., Downes T., Jones T.H. (2002). Bio-Available testosterone levels fall acutely following myocardial infarction in men: Association with fibrinolotic factors. Endocr. Res..

[13] Tripathi Y., Hegde B.M. (1998). Serum estradiol and testosterone levels following acute myocardial infarction in men. Indian J. Physiol. Pharmacol..

[14] Kumar R.G., DiSanto D., Awan N., Vaughan L.E., Levochkina M.S., Weppner J.L., Wright D.W., Berga S.L., Conley Y.P., Brooks M.M. (2020). Temporal acute serum estradiol and tumor necrosis factor-α associations and risk of death after severe traumatic brain injury. J. Neurotrauma.

[15] Chen J., Liu Y., Pan D., Xu T., Luo Y., Wu W., Wu P., Zhu H., Li D. (2022). Estrogen inhibits endoplasmic reticulum stress and ameliorates myocardial ischemia/reperfusion injury in rats by upregulating SERCA2a. Cell Commun. Signal..

[16] De Almeida S.A., Claudio E.R.G., Mengal V., Brail G.A., Merlo E., Podratz P.L., Graceli J.B., Gouvea S.A., de Abreu G.R. (2018). Estrogen therapy worsens cardiac function and remodeling and reverses the effects of exercise training after myocardial infarction in ovariectomized female rats. Front. Physiol..

[17] Separham A., Ghaffari S., Sohrabi B., Aslanabadi N., Bavil M.H., Lotfollahi H. (2017). Association of admission testosterone level with ST-segment resolution in male patients with ST-segment elevation myocardial infarction undergoing primary percutaneous coronary intervention. Basic Clin. Androl..

[18] Dong M., Mu N., Ren F., Sun X., Li F., Zhang C., Yang J. (2014). Prospective study of effects of endogenous estrogens on myocardial no-reflow risk in postmenopausal women with acute myocardial infarction. Interven. Cardiol..

[19] Ueda H., Hayashi T., Tsumura K., Kaitani K., Yoshimaru K., Nakayama Y., Yoshiyama M. (2007). QT dispersion and prognosis after coronary stent placement in acute myocardial infarction. Clin. Cardiol..

[20] Vink A.S., Postema P.G. (2021). The role of testosterone and gonadotropins in arrhythmogenesis. J. Am. Heart. Assoc..

[21] Naderi B., Yee L., Shirin S., Prior J.C., Cheung C., Davies B., Krahn A.D. (2025). Ovulatory and anovulatory cycle phase influences on QT interval dynamics during the menstrual cycle. PLoS ONE.

[22] Mouliou D.S. (2023). C-reactive protein: Pathophysiology, diagnosis, false test results and a novel diagnostic algorithm for clinicians. Diseases.

[23] Cirrincione L.R., Crews B.O., Dickenson J.A., Krasowski M.D., Rongitch J., Imoborek K.L., Goldstein Z., Greene D.N. (2022). Oral estrogen leads to falsely low concentrations of estradiol in a common immunoassay. Endocr. Connect..

[24] Fraley A.E., Tsimikas S. (2006). Clinical applications of circulating oxidized low-density lipoprotein biomarkers in cardiovascular disease. Curr. Opin. Lipidol..

[25] Jing J., Ding N., Wang D., Ge X., Ma J., Ma R., Huang X., Jueraitetibaike K., Liang K., Wang S. (2020). Oxidized LDL inhibits testosterone biosynthesis by affecting mitochondrial function and the p38 MAPK/COX-2 signaling pathway in Leydig cells. Cell Death Dis..

[26] Weitzel J.M., Vernunft A., Krüger B., Plinski C., Viergutz T. (2014). LOX-1 regulates estrogenesis via intracellular calcium release from bovine granulosa cells. Cytometry.

[27] Hartley A., Shun-Shin M., Caga-Anan M., Rajkumar C., Nowbar A.N., Foley M., Francis D.P., Haskard D.O., Khamis R.Y., Al-Lamee R.K. (2021). The placebo-controlled effect of percutaneous coronary intervention on exercise induced changes in anti-malondialdehyde-LDL antibody levels in stable coronary artery disease: A substudy of the ORBITA Trial. Front. Cardiovasc. Med..

[28] Ostinelli G., Laforest S., Denham S.G., Gauthier M., Drolet-Labelle V., Scott E., Hould F.-S., Marceau S., Homer N.Z.M., Bégin C. (2022). Increased adipose tissue indices of androgen catabolism and aromatization in women with metabolic dysfunction. J. Clin. Endocrinol. Metab..

[29] Martínez-Chacón G., Yatkin E., Polari L., Dinç D.D., Peuhu E., Hartiala P., Saarinen N., Mäkelä S. (2021). CC chemokine ligand 2 (CCL2) stimulates aromatase gene expression in mammary adipose tissue. FASEB J..

[30] Maggio M., Ceda G.P., Lauretani F., Bandinelli S., Metter E.J., Artoni A., Gatti E., Ruggiero C., Guralnik J.M., Valenti G. (2009). Estradiol and inflammatory markers in older men. J. Clin. Endocrinol. Metab..

[31] Martínez-Chacón G., Brown K.A., Docanto M.M., Kumar H., Salminen S., Saarinen N., Mäkelä S. (2018). IL-10 suppresses TNF-α-induced expression of human aromatase gene in mammary adipose tissue. FASEB J..

[32] Nasiri-Ansari N., Spilioti E., Kyrou I., Kalotychou V., Chatzigeorgiou A., Sanoudou D., Dahlman-Wright K., Randeva H.S., Papavassiliou A.G., Moutsatsou P. (2022). Estrogen receptor subtypes elicit a distinct gene expression profile of endothelial-derived factors implicated in atherosclerotic plaque vulnerability. Int. J. Mol. Sci..

[33] Nakamura Y., Suzuki T., Sasano H. (2005). Estrogen actions and in situ synthesis in human vascular smooth muscle cells and their correlation with atherosclerosis. J. Steroid Biochem. Mol. Biol..

[34] Infante M., Pieri M., Lupisella S., D’Amore L., Bernardini S., Fabbri A., Iannetta M., Andreoni M., Morello M. (2021). Low testosterone levels and high estradiol to testosterone ratio are associated with hyperinflammatory state and mortality in hospitalized men with COVID-19. Eur. Rev. Med. Pharm. Sci..

[35] Spratt D.I., Morton J.R., Kramer R.S., Mayo S.W., Longcope C., Vary C.P.H. (2006). Increases in serum estrogen levels during major illness are caused by increased peripheral aromatization. Am. J. Physiol. Endocrinol. Metab..

[36] Spratt D.I., Longcope C., Cox P.M., Bigos S.T., Wilbur-Welling C. (1993). Differential changes in serum concentrations of androgens and estrogens (in relation with cortisol) in postmenopausal women with acute illness. J. Clin. Endocrinol. Metab..

[37] Takeshita K., Hayashi M., Iino S., Kondo T., Inden Y., Iwase M., Kojima T., Hirai M., Ito M., Loskutoff D.J. (2004). Increased expression of plasminogen activator inhibitor-1 in cardiomyocytes contributes to cardiac fibrosis after myocardial infarction. Am. J. Pathol..

[38] Folsom A.R., Golden S.H., Boland L.L., Szklo M. (2005). Association of endogenous hormones with C-reactive protein, fibrinogen, and white blood count in postmenopausal women. Eur. J. Epidemiol..

[39] Rosca A.E., Vlădăreanu A., Mititelu A., Popescu B.O., Badiu C., Căruntu C., Voiculescu S.E., Onisâi M., Gologan Ş., Mirica R. (2021). Effects of exogenous androgens on platelet activity and their thrombogenic potential in supraphysiological administration: A literature review. J. Clin. Med..

[40] Maugeri N., Manfredi A.A., Maseri A. (2010). Clinical and experimental evidences on the prothrombotic properties of neutrophils. Srp. Arh. Celok. Lek..

[41] Glintborg D., Sidelmann J.J., Altinok M.L., Mumma H., Andersen M. (2015). Increased thrombin generation in women with polycystic ovary syndrome: A pilot study on the effect of metformin and oral contraceptives. Metabolism..

[42] Sowers M.R., Jannausch M., Randolph J.F., McConnell D., Little R., Lasley B., Pasternak R., Sutton-Tyrrell K., Matthews K.A. (2005). Androgens are associated with hemostatic and inflammatory factors among women at the mid-life. J. Clin. Endocrinol. Metab..

[43] Penell J.C., Kushnir M.M., Lind L., Bergquist J., Bergquis J., Lind P.M., Naessen T. (2021). Concentrations of nine endogenous steroid hormones in 70-year-old men and women. Endocr. Connect..

[44] Longcope C., Pratt J.H., Schneider S.H., Fineberg S.E. (1978). Aromatization of androgens by muscle and adipose tissue in vivo. J. Clin. Endocrinol. Metab..

[45] Qui X., Zhang H., Ye D., Yao X., Zhang S., Dai B. (2013). Variations in circulating sex steroid levels in metastatic prostate cancer patients with combined androgen blockade: Observation and implication. Andrology..

[46] Gautier A., Bonnet F., Dubios S., Massart C., Grosheny C., Bachelot A., Aubé B., Balkau B., Ducluzeau P.-H. (2013). Associations between visceral adipose tissue, inflammation and sex steroid concentrations in men. Clin. Endocrinol..

[47] Shan B., Shao M., Zhang Q., Hepler C., Paschoal V.A., Barnes S.D., Vishvanath L., An Y.A., Jia L., Malladi V.S. (2020). Perivascular mesenchymal cells control adipose-tissue macrophage accrual in obesity. Nat. Metab..

[48] Tostes R.C., Carneiro F.S., Caryvalho M.H.C., Reckelhoff J.F. (2015). Reactive oxygen species: Players in the cardiovascular effects of testosterone. Am. J. Physiol. Regul. Integr. Comp. Physiol..

[49] Mondragonn R.R., Wang S., Stevenson M.D., Lozhkin A., Vendrov A.E., Isom L.L., Runge M.S., Madamanchi N.R. (2025). NOX4-driven mitochondrial oxidative stress in aging promotes myocardial remodeling and increases susceptibility to ventricular tachyarrhythmia. Free Radic. Biol. Med..

